# Detecting Differences of Fluorescent Markers Distribution in Single Cell Microscopy: Textural or Pointillist Feature Space?

**DOI:** 10.3389/frobt.2020.00039

**Published:** 2020-05-22

**Authors:** Ali Ahmad, Carole Frindel, David Rousseau

**Affiliations:** ^1^Laboratoire Angevin de Recherche en Ingénierie des Systèmes, UMR INRAE IRHS, Université d'Angers, Angers, France; ^2^Centre de Recherche en Acquisition et Traitement de l'Image pour la Santé, CNRS UMR 5220-INSERM U1206, Université Lyon 1, INSA de Lyon, Lyon, France

**Keywords:** microscopy, fluorescence, classification, texture, spot detection, point spread function

## Abstract

We consider the detection of change in spatial distribution of fluorescent markers inside cells imaged by single cell microscopy. Such problems are important in bioimaging since the density of these markers can reflect the healthy or pathological state of cells, the spatial organization of DNA, or cell cycle stage. With the new super-resolved microscopes and associated microfluidic devices, bio-markers can be detected in single cells individually or collectively as a texture depending on the quality of the microscope impulse response. In this work, we propose, via numerical simulations, to address detection of changes in spatial density or in spatial clustering with an individual (pointillist) or collective (textural) approach by comparing their performances according to the size of the impulse response of the microscope. Pointillist approaches show good performances for small impulse response sizes only, while all textural approaches are found to overcome pointillist approaches with small as well as with large impulse response sizes. These results are validated with real fluorescence microscopy images with conventional resolution. This, a priori non-intuitive result in the perspective of the quest of super-resolution, demonstrates that, for difference detection tasks in single cell microscopy, super-resolved microscopes may not be mandatory and that lower cost, sub-resolved, microscopes can be sufficient.

## 1. Introduction

Over the last two decades, microscopy benefited from several scientific revolutions. For instance, innovations in chemistry via the production of new fluorescent markers, in optics with lasers tunable both in wavelength and impulse duration, or innovations in microfluidic bringing *in vitro* samples under the microscope automatically. These revolutions enabled the advent of intermediate to super-resolution microscopy techniques, such as lattice light sheet fluorescence microscopy (LLSFM), structured illumination microscopy (SIM), stimulated emission depletion microscopy (STED), or single molecule localization microscopy (PALM/STORM) techniques (Betzig et al., 2006; Rust et al., 2006; Schermelleh et al., 2010; Stelzer, 2015; Cremer et al., 2017). It is now possible to observe in 2D or 3D sub-cellular items inside single cells with resolutions which goes below the Rayleigh criterion for a classical microscope (Lakadamyali and Cosma, 2015; Ryabichko et al., 2017). These super-resolved systems are still not fully transferred in industrial applications or even in microscopy platforms open to users that would not be expert in instrumentation. One reason for this translation delay is that super-resolution comes with the price of constraints in terms of micro-positioning which are more demanding as the size of the point spread function of the microscope is smaller. One way to relax such constraints consists in coupling the choice of the optical elements, i.e., designing the point spread function, jointly with the biological question raised and the associated image processing pipelines. We propose such an approach in this work.

**Figure 1:**
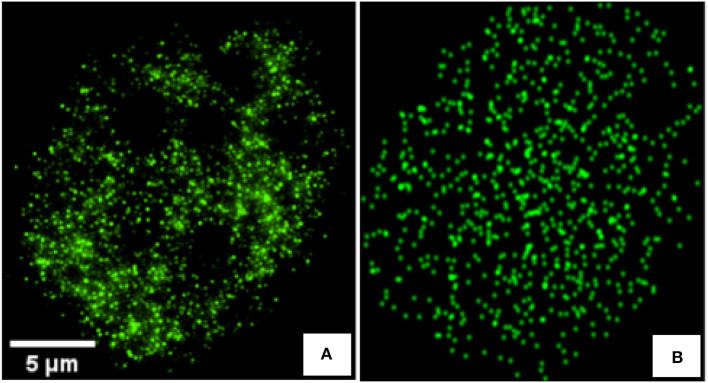
**(A)** Real 2D image of a C2C12 cancerous cell immunostained using Alexa Fluor 488 antibodies and acquired with a N-SIM super-resolution microscope system (Nikon Instruments) equipped with CFI Apo TIRF 100× 1.49 N.A oil immersion objective. **(B)** Synthetic image, generated to mimic image of **(A)**, with an heterogeneous Poisson distribution of markers and convolved by a gaussian kernel of σ_*psf*_ = 0.8 simulating the PSF.

**Figure 2:**
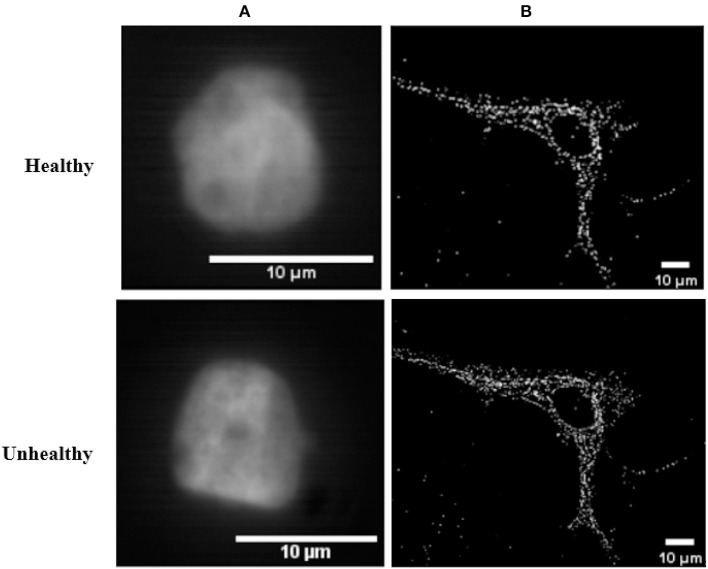
Real 2D images of healthy and unhealthy nuclei of cells from different microscopy techniques. **(A)** Nuclei of breast cells images acquired by an Aurox Clarity structured illumination/spinning disk laser-free confocal microscopy system. The lens used was a 63× 1.4NA Oil objective, with for a 583 nm excitation and 631 nm emission wavelengths, and looking at mCherry labeled histone-H2B. **(B)** Nucleoids of skin fibroblast mitochondria stained with fluorescein isothiocyanate (FITC) and acquired with super-resolved stochastic optical reconstruction microscopy technique.

In this article, we consider images of single cells observed with a microscope in which fluorescent markers have been activated (see Figure 1). We consider informative tasks that consist in detecting differences in the spatial organization of these fluorescent markers. Such differences could be either in terms of density or in terms of clustering. Detecting changes in spatial organization tasks are important issues in numerous biological contexts. For instance, distinct epigenetic states are associated with specific chromatin spatial modifications and compactions. Hence, defining the 3D-organization of cancer-associated chromatin domains could represent a new frontier to decipher tumor heterogeneity during tumor progression and metastasis formation (Kundu et al., 2017; Boettiger et al., 2016; Stevens et al., 2017). In another instance, the detection of nucleoids distribution changes is an important issue for the study of mitochondrial defect under various stresses. For example, disturbance in nucleoids components and mutations in mtDNA were identified as significant in various diseases, like carcinogenesis (Lee and Han, 2017) and neurodegenerative diseases (Chevrollier et al., 2012). These two use-cases focused on chromatin or on nucleoids of mitochondria are illustrated in Figure 2. The binary classification task here corresponds to a detection between healthy and unhealthy from the observation of the fluorescent markers inside individual cells. The use-cases illustrated in Figure 2 were produced with a sub-resolved microscope in panel A where markers appear as a texture and are not distinguishable from one another while it was produced with a super-resolved microscope in panel B where markers can all be located individually. However, to achieve the global characterization of a cell, it might not be necessary to locate individually each of these markers and thus, there is no guaranty that super-resolution is indeed mandatory. Such considerations are very important in practice because super-resolved microscopes are much more costly than sub-resolved microscopes in terms of optics, acquisition procedure, or numerical memory load. It would therefore be very useful to be able to determine a priori what would be the best resolution for a given task in order to choose the most appropriated microscope or design an optimal point spread function (PSF). In practice, for experimental optical acquisition, several acquisition conditions could be tested offering various PSF sizes. In simulation, a continuous set of PSF can be tested freely offering a complete view of how the cell detection would behave and enable to envision what would be the good range of PSF before real implementation.

Two main families of approaches are proposed in the literature to address the characterization of cells in sub-resolved microscopy (Kleppe et al., 2018; Paunovic et al., 2019) and super-resolved microscopy Griffié et al. (2016), Xu and Liu (2019). For the sub-resolved case, where cells are determined by more or less organized global patterns, one can use a textural approach. While for the super-resolved case, fluorescent markers distributions are classically studied with a pointillist approach. We propose to compare, for the first time to the best of our knowledge, the performances of algorithms based on the identification of each markers (pointillist approach) or the characterization of the texture created by these markers (textural approach). We compare the performances of these algorithms for different sizes of point spread function of a microscope and specially focus on the situations where the optical systems passes from super-resolved to sub-resolved. Such an experiment would be very time consuming to be undertaken with real microscopes and can benefit from a simulation scheme as proposed here and also as current practice in the literature (Lehmussola et al., 2007; Rubin-Delanchy et al., 2015; Gazagnes et al., 2017; Samacoits et al., 2018; Ma et al., 2019). Simulated images are produced with the help of simulated point spread functions which realistically mimics real fluorescent images as shown in Figure 1B. An example of real images is also provided to validate the result obtained on simulation.

The article is organized in the following way. The process for the simulation of the images is first given. Then tools used for the characterization with textural or pointillist approaches are described. The comparison of these feature spaces reduced to the same dimension and applied to the same classifier is then produced before discussion and conclusion.

## 2. Simulating Fluorescence Microscopy Images of Single Cells

Two simulations were realized to investigate two distinct binary classifications with a difference in fluorescent markers density or a difference in spatial clustering of fluorescent markers. This corresponds to the practical situation of cases illustrated in Figure 2.

**Figure 3:**
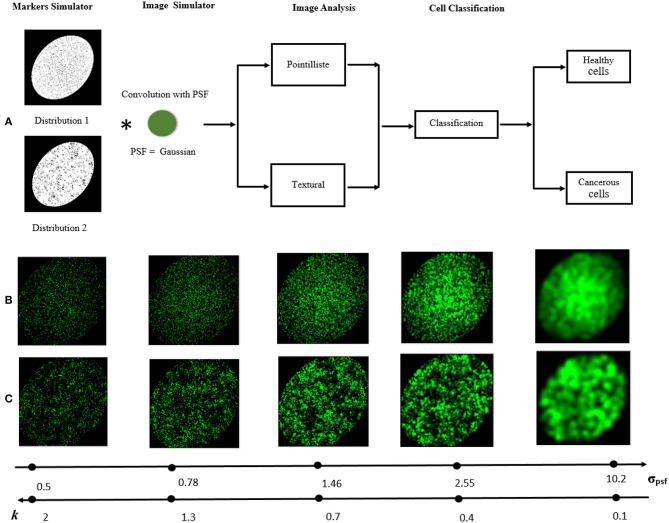
Visual abstract. **(A)** The processing pipeline to simulate cell and classify them with distinct fluorescent marker densities or organizations. The image acquired by the microscope is simulated by the convolution of the distribution of markers with the PSF. To classify the cell types, two approaches are studied for different values of PSF: pointillist and textural. **(B,C)** Examples of synthetic microscopic images [(*C*_1_) or (*C*_2_) in **(B)** and (*C*_3_) in **(C)**] for different values of σ_*psf*_ and the corresponding *k* ratio according to Equation (1).

First, we generated two populations of cells (*C*_1_) and (*C*_2_) with a difference of marker density. The coordinates (*x, y*) of each fluorescent marker were picked randomly according to independent and identically distributed Gaussian distributions on horizontal and vertical dimensions of an image of *M* × *N* pixels, respectively, where *x* ∈ {1, 2, 3, …, *M* = 256} and *y* ∈ {1, 2, 3, …, *N* = 256}. The two classes of cells, mimicking healthy (*C*_1_) and pathological (*C*_2_), were generated with a difference of standard deviation in their distributions (Figures 3A,B). The parameters were empirically adjusted to mimic observations on the real cells of Figure 1A, with for healthy cells (*C*_1_) : *N*_*x*_(126, 100), *N*_*y*_(126, 100), a total amount of markers of 3, 000, the area of the cell is 100 × 100 = 10, 000 pixels, and a resulting density of markers $\frac{number\;of\;markers}{area\;of\;the\;cell}=0.3$. For the pathological cells (*C*_2_) we have *N*_*x*_(126, 100), *N*_*y*_(126, 90), a total number of markers of 3, 000, an area of 100 × 90 = 9, 000 pixels, and a resulting density of markers of 0.33. The difference of marker density between classes is of 0.03. We used here this difference between (*C*_1_) and (*C*_2_) to compare the pointillist and textural approaches.

Second, we generated populations of cells with same density of markers but with different spatial organization. We considered the task to detect the difference between cells with heterogeneous Poisson processes markers like (*C*_1_) and cells with clustered markers that we coined (*C*_3_). Clustered fluorescent markers were generated with a two steps process (i) 300 seeds were, independently from each other, distributed uniformly on horizontal and vertical space of the image then (ii) around each generated seed *s* ∈ {1, 2, 3, …, 300}, 10 markers were generated with a distance to the seed which follows an exponential distribution *D*_*s*_ $(0,\;\lambda_{D}=\frac{1}{\mu_{D}}$), where λ_*D*_ is the rate parameter of this distribution and μ_*D*_ is the mean of the distribution which was set to 35 (Diggle, 1983). The overall number of markers generated in each image was 3, 000. An instance of this cell class *C*_3_ is shown in Figures 3A,C. Clustered (*C*_3_) and heterogeneous Poisson processes markers (*C*_1_) organization was used to compare the pointillist and textural approach. A crop inside the simulated cells is performed to avoid any issue concerning on the boundary of the simulated cells.

The PSF of the microscope was modeled by a convolving kernel here taken for illustration as a Gaussian kernel with size σ_*psf*_. The simulated images were then simply the convolution of the randomly and clustered positioned fluorescent dots with the PSF (see Figure 3). Each image was 256 × 256 pixels and the realism can visually be appreciated in Figures 1A,B. The performance of the classification between two types of cells ((*C*_1_) (*C*_2_)) or ((*C*_1_) (*C*_3_)) was investigated as a function of the size of the PSF governed by σ_*psf*_. This situation corresponds to the practical use-case in instrumentation where ones seeks to design the PSF of a microscope for a given informative task. Here, the objective was to find the condition of PSF which enables to obtain the best binary classification performance.

The range of exploration of the size of the PSF σ_*psf*_, was adimensioned by the distance between markers

(1) $$\begin{array}{l} k=\left(\frac{d_{minC_{1}C_{2}}}{\sigma_{psf}}\right) \end{array}$$

where *d*_*minC*_1_*C*_2__ is the smallest value of the minimal distances between markers calculated for *C*_1_ and *C*_2_. This distance computed for the simulated data set is *d*_*minC*_1_*C*_2__ = 1.02. We have explored the values of σ_*psf*_ around *k* = 1 which intuitively corresponds to the switch between the super-resolved regime (*k* > 1) to the sub-resolved (*k* < 1) as shown in Figure 3C.

As a complement to simulation, the same approach was applied to a real data set consisting of healthy and unhealthy (cancerous) breast single cells. These reals images shown in Figure 2A can be considered to be in the sub-resolved regime due to the estimated large experimental PSF of the microscope. The total amount of the real data set is 907 cancerous cell images and 1, 007 healthy cell images.

## 3. Pointillist Feature Spaces

A first step before studying fluorescent markers spatial distribution, is to localize them. In the literature, several algorithms for the localization of fluorescent markers have been developed (Holden et al., 2011; Ovesný et al., 2014; Gazagnes et al., 2017). For our study, we used UNLOC (*Unsupervised particle localization*), the state of the art method recently introduced in Mailfert et al. (2018).

**Figure 4:**
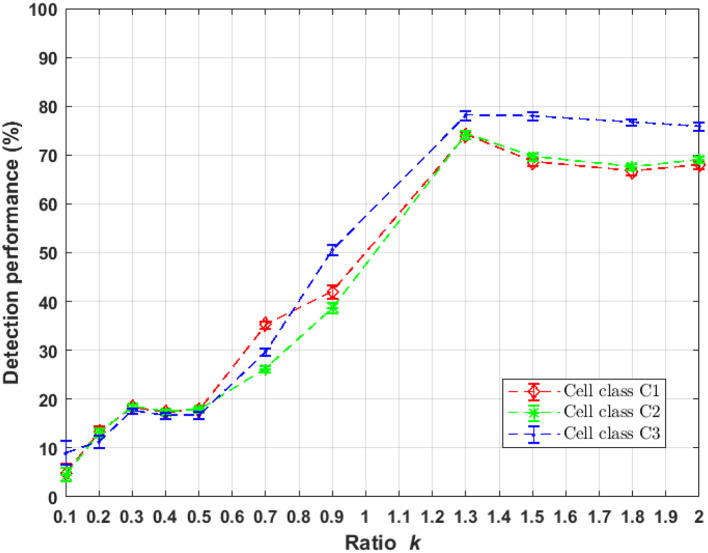
Performance of markers localization using UNLOC in randomly and clustered distribution as a function of parameter *k* of Equation (1). UNLOC is more efficient in the range *k* = [1.3 2].

UNLOC is a fast algorithm free of parameter that provides a list of coordinates and associated parameters for each detected particle for *a posteriori* quantification and image reconstruction. The algorithm is based on the decision theory without the need of initialization of any parameters relative to the data (SNR, particle density, background level). Only parameters relative to the optical setup must be provided like the PSF size (σ_*psf*_) of the microscope to perform a PSF-deconvolution step. UNLOC has been shown in Mailfert et al. (2018) to approach the Cramér-Rao bound for the detection of particles in high density and without prior knowledge of their intensity. We applied UNLOC to the simulated images in sub-resolved and super-resolved regimes. Markers detection performance is presented in Figure 4. A uniform increase of detection performance for both randomly distributed markers with heterogeneous Poisson processes and clustered markers organization occurs for a *k* = [0.5 1.3] which corresponds to a PSF size σ_*psf*_ < 2. A maximum performance of around 80% of detected markers occurs for *k* = 1.3 corresponding to σ_*psf*_ = 0.8 the range where UNLOC achieve a maximum performance of detection as found in Mailfert et al. (2018) where the minimum inter-marker distance is > 1.23σ_*psf*_ (*d*_*minC*_1_*C*_2__ = 1.02 > 1.23σ_*psf*_).

After extracting the markers, it is necessary to characterize their spatial organization statistically. In the following section, we describe the proposed statistical descriptors computed in the pointillist approach and introduced to detect density and organization differences of fluorescent markers.

### 3.1. Distribution Analysis

**Figure 5:**
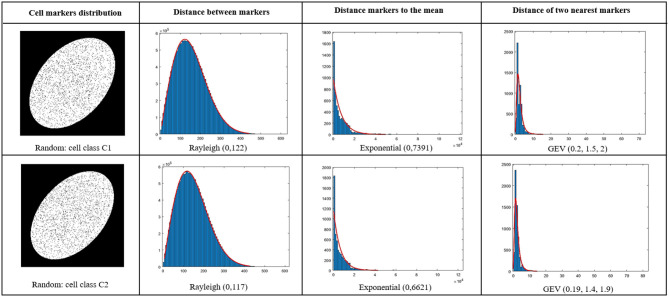
Fit of distances with distributions selected from *BIC* criterion among a large set of common distributions as in Neath and Cavanaugh (2012). Blue stands for histogram and red for the fit. Upper line for a cell (*C*_1_) and a cell (*C*_2_) on the second line.

This method was applied for marker density detection problem. We analyzed statistically the markers detected by UNLOC by computing the distance between markers, the distance of markers to the mean markers position and the distance between each marker and its nearest neighbor. From these distances, a large set of 16 common distributions of the literature were tested as proposed in Aminov (2019). As a tradeoff between the quality of the fit and the number of parameters in the distribution, the best distribution among all the one tested, was selected to minimize the bayesian inference criterion (*BIC*) (Schwarz, 1978; Neath and Cavanaugh, 2012) expressed as

(2) $$\begin{array}{l} BIC=-2ln\left(L\right)+p.ln\left(nb\right) \end{array}$$

where *L* is the likelihood of the model, *nb* the number of observations in the sample and *p* the number of parameters in the model. In our study, the selected model for each calculated distance according to *BIC* was found to be Rayleigh *R*(0, *scale σ*_*R*_) for the distance between markers, exponential exp(0, *rate λ*_*E*_) for the distance to the mean and generalized extreme value *GEV*(*shape ξ*_*G*_, *scale σ*_*G*_, *mean μ*_*G*_) for the distance of markers to the closest neighbor. Illustration of quality of these fits for the distribution parameters are given in Figure 5. These five statistical parameters (σ_*R*_, λ_*E*_, ξ_*G*_, σ_*G*_, μ_*G*_) of the distance distributions were then used as features for the classification between cells.

### 3.2. Spatial Pattern Analysis

In this work, we are interested in detecting the differences between classes of cells based on the change in spatial organization of fluorescent markers. For this aim, we work with Ripley's K-function that is classically used in microscopy (Hansson et al., 2013; Amgad et al., 2015; Samacoits et al., 2018) to summarize completely spatial randomness or clustering behavior of fluorescent markers and estimate markers organization parameters. But, in our work, we used this function as a descriptor to detect change between heterogeneous Poisson processes and clustered distributed markers. By definition, Ripley's K-function is a spatial analysis to describe how point patterns occur over a given area of interest (circle of radius *r*) and whose standard expression is

(3) $$\begin{array}{l} K\left(r,n\right)=\frac{\left|\Omega \right|}{n\left(n-1\right)}\underset{x\neq y}{\sum }L_{\left\{|x-y|\leq r\right\}}f\left(x,y\right) \end{array}$$

where *n* is the total number of events within the given field of view |Ω|, *L*_{|*x*−*y*|≤*r*}_ is an indicator function equal to 1 if the distance between markers located in *x* and *y* is smaller than the radius *r*, and equal to 0 otherwise. *f*(*x, y*) is a boundary correction term that prevents a bias in *K*(*r, n*) at large values of *r* due to the finite size of |Ω|. Indeed, some pairs of markers closer than *r* can fall outside the observation window |Ω|, leading to an underestimation of *K*. Multiple edge correction methods have been devised for Ripley's K-function. The most widely used boundary correction is the Ripley's correction $f\left(x,y\right)=\frac{1}{2}\left(P\left(x,y\right)+P\left(y,x\right)\right)$, where $P\left(x,y\right)=\frac{\left|\partial b\left(x,|x-y|\right)\right|}{\left|\partial b\left(x,|x-y|\right)\cap \Omega \right|}$. It consists of dividing the number of events at a certain distance from the central event by the proportion of the circumference of a circle ∂*b*(*x*,|*x*−*y*|) that is included within the field of view |Ω| (Lagache et al., 2013). So with this boundary correction and under the hypothesis of completely random process, the expectation 𝔼[*K*(*r, n*)] = π*r*^2^ (Ripley, 1991, page 39). One problem with the original Ripely's *K*-function is that it is not centered and normalized which complicates its quantitative interpretation. In our work, we used the estimation version proposed by Besag (1977) given as

(4) $$\begin{array}{l} \hat{K}\left(r,n\right)=\sqrt{\frac{K\left(r,n\right)}{\pi }}-r. \end{array}$$

**Figure 6:**
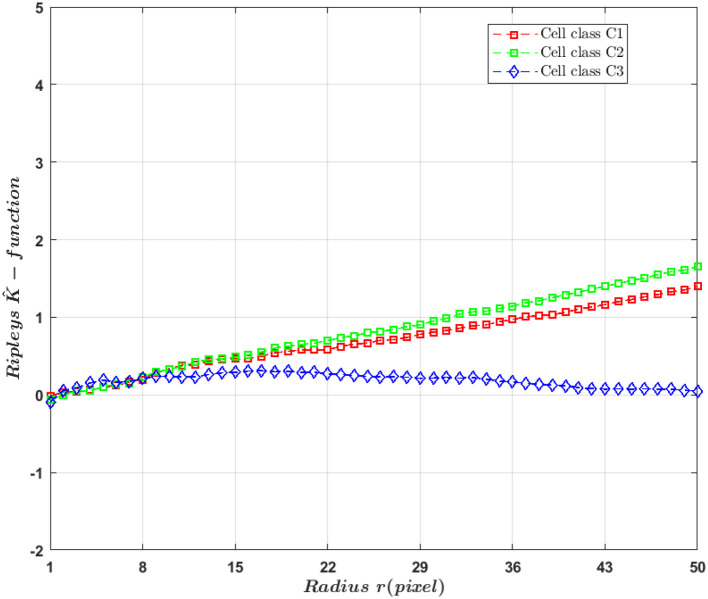
$\hat{K}\left(r,n\right)$ curve for raw markers (i.e., non-convolved with a PSF) distribution according to radius *r*.

In this work, we exploited $\hat{K}$ curve differences between the studied cases (see Figure 6) for the detection tasks between class of cells with markers density differences (*C*_1_ and *C*_2_) and spatial distribution differences (*C*_1_ and *C*_3_). We extracted from the $\hat{K}$ curves five features: the maximum $\hat{K}$ value, the maximum gradient [0, *max*], the minimum gradient [*max, end*], the radius corresponding to the maximum $\hat{K}$ value and Spearman correlation between $\hat{K}$ and the radius *r* similarly to what was proposed in Samacoits et al. (2018).

## 4. Textural Feature Spaces

In this section, we describe the textural methods used to detect a difference of fluorescent markers density or a difference of spatial clustering of fluorescent markers. There is a wide range of methods (Mirmehdi, 2008) and there is no proof of optimality for any tool. We do not intend to be exhaustive and pick up a selection of classical methods. For a fair comparison of all tested textural and pointillist methods, the feature spaces produced by each textural method was reduced to the dimension of the method with the smallest feature space with a principal component analysis.

### 4.1. Auto-Correlation

A standard tool to characterize the second-order statistics of a texture consists in analyzing the spatial Fourier transform of the auto-correlation of an image. This was for instance used to characterize the arrangement of fluorescent markers in microscopy (Kolin and Wiseman, 2007; Robertson and George, 2012). By definition, auto-correlation is equivalent to comparing all possible pixel pairs and reporting the likelihood that both will be bright as a function of the distance and direction of separation. Mathematically, auto-correlation is the convolution of a function with itself. For a microscopy image *I* of size *M* × *N*, auto-correlation function *G*(*a, b*) is calculated by the following equation

(5) $$\begin{array}{l} G\left(a,b\right)=\overset{M}{\underset{x}{\sum }}\overset{N}{\underset{y}{\sum }}i\left(x,y\right)*i\left(x-a,y-b\right) \end{array}$$

where *i*(*x, y*) is the image intensity at position (*x, y*) and *a* and *b* represent the shift from the initial position *x* and *y*. Practically, auto-correlations is calculated more efficiently and in a quicker way via fast Fourier transforms using the Wiener-Khinchin theorem stating that the auto correlation of an image is equal to the Fourier transform (*F*) of the power spectrum of this image. In our study, the used auto-correlation computation method was as the following

(6) $$\begin{array}{l} G\left(i\right)=F^{-1}\left[PS\left(i\right)\right] \end{array}$$

where *PS*(*i*) = |*F*[*i*(*x, y*)]|^2^ is the power spectrum of the image. The shape of the auto-correlation can be summarized with various features. In our study we computed five features for our classification tasks: maximum auto-correlation value, full width at half maximum (FWHM), maximum and minimum gradient and the variance the remaining portion of the autocorrelation functions profile after removing the central peaks.

### 4.2. Gray Level Co-occurrence Matrix (GLCM)

Another classical statistical approach that can well-describe second-order statistics of a texture image is provided by the so-called gray level co-occurrence matrix (GLCM). GLCM was firstly introduced by Haralick et al. (1973) and is essentially a two-dimensional histogram in which the (*i, j*)*th* element is the frequency of pixel intensity *i* co-occurring with pixel intensity *j*. A co-occurrence matrix is specified by the relative frequencies *C*(*i, j, d*, θ) in which two pixels, separated by a distance *d*, occurs in a direction specified by the angle θ, one with gray level *i* and the other with gray level *j*. A set of 14 Haralick coefficients summarizing the GLCM is then computed. In our study, since we expected no specific orientation a priori, and, as a trade off to respect this isotropy and limit the computation time, we included four directions for θ: 0, 45, 90, and 135°. The size of the neighborhood was chosen to be a multiple of the maximum value of σ_*psf*_, and found optimal at 72 × 72 pixels (see Supplementary Material section 2). A principal component analysis was then applied to select only the five first significant components from the 14 Haralick coefficients. The five most significant features for the whole range of tested PSF size were found to be contrast, variance, sum variance, difference variance, and sum average.

### 4.3. Local Binary Patterns (LBP)

Local binary patterns are also among the most used texture descriptors in classification tasks (Ojala et al., 2002). In our study, the LBP was computed by dividing each original microscopy image in regions of 72 × 72 pixels similarly to the scale chosen with the GLCM. For each central pixel position coordinate (*x, y*) of these regions, local binary pattern (LBP) indicates a sequential set of the binary comparison of its value with the eight neighbors. So that the LBP assign to each neighbor the value 0 or 1, if its value is smaller or greater than the pixel placed at the center, respectively. The resulting decimal value of the generated binary number replaces the central pixel value and can be expressed as follows

(7) $$\begin{array}{l} LBP\left(x,y\right)=\overset{7}{\underset{n=0}{\sum }}2^{n}b\;\left(i_{n}-i_{x,y}\right) \end{array}$$

where *i*_*x,y*_ is the gray value of the central pixel and *i*_*n*_ denotes the *n*th neighboring one. Besides, the function *b*(*z*) is defined as follows

(8) $$\begin{array}{l} b\left(z\right)=\{\begin{array}{l} 1,\text{ if }z\geq 0\\ 0,\text{ if }z<0. \end{array} \end{array}$$

The frequency of occurrences of each decimal code was then calculated over each region and used as a texture descriptor. A principal component analysis was finally applied to reduce the total number of descriptors per image to 5 as in the GLCM approach.

## 5. Classification

Classification tasks were addressed to discriminate between cells populations ((*C*_1_) and (*C*_2_)) with a small difference of fluorescence marker density and between cells ((*C*_1_) and (*C*_3_)) with different spatial marker organizations. For fair comparison all features spaces either pointillist and textural was set to 5. These features spaces were applied to the same simple support vector machine with linear kernel. Comparison with other classical classifiers (decision tree, logistic regression classifier, and K-nearest neighbors) are also provided in the Supplementary Material. The classification performance was tested for 11 different values of σ_*psf*_ ranging from sub-resolved to super-resolved regimes. For each value of σ_*psf*_ the simulated data set was composed of 4, 000 images for training, with 2, 000 for each class, and 500 images for test (respectively 250 images for each class). Classification was also performed on the real data set of Figure 2A. Standard deviation of performances were computed using 10-folds cross-validation method.

## 6. Results

### 6.1. Difference of Density

**Figure 7:**
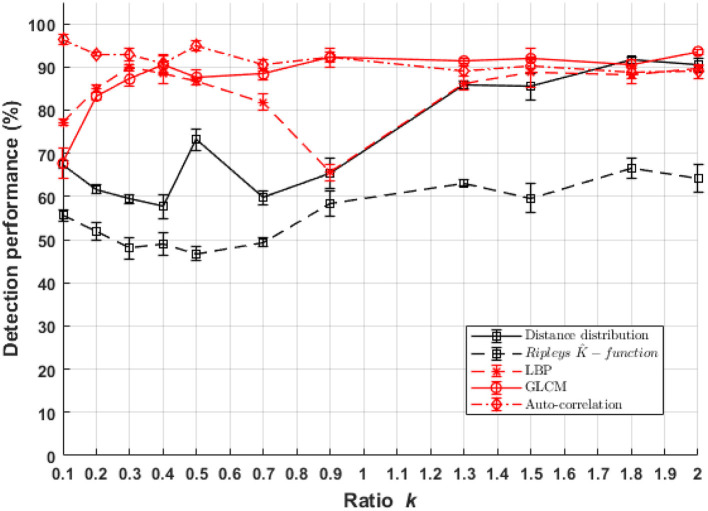
Performance of binary classification for marker density difference between cell classes *C*_1_ and *C*_2_ as a function of parameter *k* of Equation (1) for both textural and pointillist approaches. The higher *k* the smaller the PSF size. Standard deviation of performances are computed using 10-folds cross-validation method. In red, methods following the textural approach, and in black, methods following a pointillist approach.

Classification performances between populations of cells with a small fluorescent marker density differences ((*C*_1_),(*C*_2_)) as a function of σ_*psf*_ are presented in Figure 7. The performance of the textural approach overpasses everywhere the pointillist ones either in sub-resolved and also super-resolved regimes. Among the textural features spaces, auto-correlation textural approach shows the most stable classification performance for all PSF sizes. Distance distribution among the pointillist approaches shows good performances for the detection of difference in the super-resolved regime only. Since we did not test all existing methods for the pointillist and the textural approaches, comparison is not exhaustive. Nevertheless, one should here recall and underline the specific choices for the tested methods (Haralick coefficient, Local binary pattern and Auto-correlation) which all constitute very basic methods for the textural approach. Therefore, other textural approaches could surely provide even better results while the UNLOC method for dense detection of fluorescence has been shown to be the current reference for the state of the art (Mailfert et al., 2018). We can thus conclude that, globally, for the considered classification task, feature spaces based on textural approaches outperform a pointillist-based feature space and this in both sub-resolved and super-resolved regimes.

It should be noticed that there is not only a difference in density between (*C*_1_) and (*C*_2_) but also in spatial organization because of the different standard deviations used for the simulation. This is why a differences between cell classes *C*_1_ and (*C*_2_) was found in Ripely's *K*-function (see Figure 6). If the only difference between classes were in the density, then theoretically they would be no difference in *K*-function. It is indeed a basic design property of this descriptor to capture only second-order characteristics of a point process, and to be invariant to changes in density. A variant of the simulation of classes (*C*_1_) and (*C*_2_) with differences only based on density without the diffraction effect of the microscope were conducted in the Supplementary Material. As expected the detection of difference of density with Ripley's *k*- function is pointless for the super-resolved cases. However, due to the convolution process and to the instability of the UNLOCK detection which strongly depends on σ_*psf*_ some discriminant effect can occur between cell classes (*C*_1_) and (*C*_2_) in the sub-resolved cases. The simulation details and results for this experiment are described in Supplementary Material, section 3.

### 6.2. Difference of Spatial Organization

**Figure 8:**
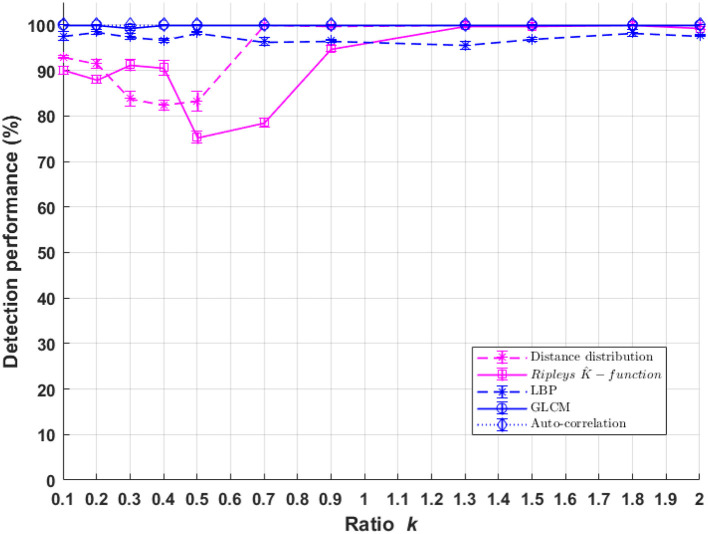
Performance of binary classification for markers spatial organization difference for cell classes *C*_1_ and*C*_3_ as a function of parameter *k* of Equation (1) for both textural and pointillist approach. The higher *k* the smaller the PSF size. Standard deviation of performances are computed using 10-folds cross-validation method. In blue, methods following the textural approach, and in pink, methods following a pointillist approach.

**Figure 9:**
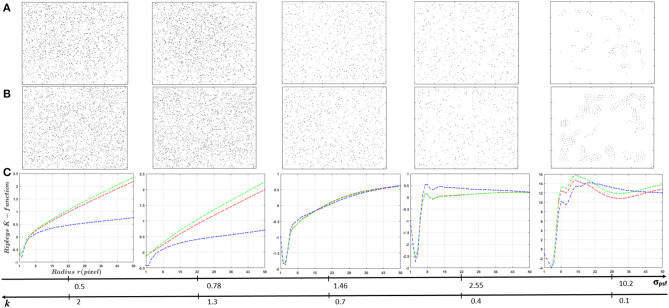
Influence of PSF size (ratio *k*) on Ripley's K-functions $\hat{K}\left(r,n\right)$ of localized markers using UNLOC. **(A)** Visualization of localized markers in random distribution case. **(B)** Visualization of localized markers in clustered distribution case. **(C)** $\hat{K}\left(r,n\right)$ curves. Spatial organization of fluorescent markers changes according to the PSF size. Globally, for small PSF sizes (super-resolution images), markers organizations shows a similar organization as the raw data (Figure 6). Switching to sub-resolution images (large PSF size), spatial organization of detected markers changes due to miss detection caused by UNLOC sensitivity drop.

Classification performances between populations of cells with a difference of fluorescent marker spatial distribution ((*C*_1_),(*C*_3_)) as a function of σ_*psf*_ are presented in Figure 8. The performance of the textural and pointillist classification approaches are found to be very high and very close to each other almost everywhere in terms of σ_*psf*_ and, remarkably in the sub-resolved regimes. The sub-resolved regime, not surprisingly, is the place where the performance of the pointillist approach drops. Some markers are detected as artifacts but the spatial organization of the two populations of cells becomes very close so that the discrimination between them drops. This is illustrated in Figure 9 with the global evolution of the Ripley's K-functions when plotted for various σ_*psf*_.

### 6.3. Test on Real Data

Classification performances between populations of healthy and cancerous cells of real data set from sub-resolved microscopy technique of Figure 2A are presented in Table 1. As a global comparison with the results of synthetic data, performances behaviors are globally similar. Auto correlation approach of textural feature space shows the best classification performance with an important gap with other proposed textural and pointillist feature spaces. By contrast with what was found in simulation, LBP performance is less than the GLCM performance. This may come from the fact that LBP is sensitive to the noise, such as the thermal noise of the camera. Such noise was not taken into account in the simulation. Other derivative of LBP were proposed in the literature like median binary pattern (MBP) (Hafiane et al., 2007), local ternary patterns (LTP) (Tan and Triggs, 2010), and improved LTP (ILTP) (Kylberg and Sintorn, 2013). These could be tested to circumvent this noise sensitivity problem. Another important point to assess the value of a feature space lay in its computational time. They are presented in Table 2. A tradeoff between performances and computational time is found with the correlation based textural feature space.

**Table 1:** Classification results of the studied methods applied to real data set of Figure 2A.

**Feature spaces**	**Real data**
Auto-correlation	94.5 ± 0.18 %
GLCM	70 ± 1.4 %
LBP	64 ± 1.4 %
Distance distribution	78.8 ± 0.7 %
Ripley's $\hat{K}$-function	69 ± 1.7 %

**Table 2:** Features space computational time averaged on 100 real images.

**Feature spaces**	**Auto-correlation**	**GLCM**	**LBP**	**Distance distribution**	**Ripley's $\hat{K}$-function**
Computation time (ms)	0.66	0.12	6.3	340	68, 790

## 7. Conclusion

In this study, we have simulated two classes of images of cells (healthy and pathological) with fluorescent markers having either a weak difference of density or a difference of spatial organization. We have then simulated different size of microscope PSF around the switch of regime between super-resolved and sub-resolved of the markers. These synthetic data sets served to compare the detection performance both with a textural and a pointillist approaches. We found that the textural approach reaches better performances in all regimes sub-resolved as well as super-resolved. We also tested a real data set acquired with sub resolved microscopy. In accordance with the result on synthetic data, results from this real data set showed that the classification performance when using auto correlation textural approach overcome GLCM and LBP textural approaches as well as pointillist approaches.

Somehow counter-intuitive when considering the current quest for super-resolution, this strong and practically important results demonstrates that it may not be necessary to systematically search for expansive super-resolution techniques or perform time-consuming deconvolution when gazing at collective spatial organization of fluorescent markers in single-cell microscopy. This result is more common in signal processing. Indeed, an analogy is found in kernel-based density estimation methods where the kernel (the PSF in the case studied here) spread the information contained in discrete points to a larger area and thus contribute to create its continuous representation. This representation is easier to handle than the discrete one. Back in microscopy, when detection or classification is targeted sub-resolved images can carry sufficient information to enable high performances. This was obtained here with simulated images where the ground truth was established automatically and with one real data set. This exactly corresponds to the situation where cells can be sorted automatically based on a biological experimental plan or using standard flow cytometry. If such ground truth cannot be established, it might be the case that, similarly to what happens in histology, only a visual human inspection of the cell can enable to constitute a reference on which supervised learning can be trained. In this case, even for classification tasks, super-resolution coupled with sub-resolution may be necessary. However, if such pairs are constituted during the training, then only sub-resolution images can be used for classification during the testing as shown in this work.

Further investigations could be undertaken in at least two directions. First, in this article the PSF of the microscope was purposely naïve under the form of a simple Gaussian 2D kernel. The proposed methodology could easily be translated without any difficulty to any type of more realistic PSF and can even be extended in 3D. The global methodology could thus be used in instrumentation to validate the quality of a PSF for a given informative task. The realism of the simulator could also be enhanced to account for non-spatial invariance of the PSF due to the sample (Cuplov et al., 2014) or the non-uniformity of fluorescence intensity of the markers. Second, only binary classification tasks were considered in this article and it could be interesting to consider if other informative tasks, such as regression could benefit from the proposed approach.

## Data Availability Statement

The simulator developed for this article will be available on request after acceptance of this article.

## Author Contributions

The experiment conceptualized, curation, formal analysis, funding acquisition, investigation, methodology, project administration, resources, software, supervision, validation, visualization, writing review, and editing have been performed equally by AA, CF, and DR.

## Conflict of Interest

The authors declare that the research was conducted in the absence of any commercial or financial relationships that could be construed as a potential conflict of interest.
