# Supplementary material for: Detecting Differences of Fluorescent Markers Distribution in Single Cell Microscopy: Textural or Pointillist Feature Space?
Source: Front Robot AI. 2020 May 22;7:39. doi: 10.3389/frobt.2020.00039 (PMC7805927; doi:10.3389/frobt.2020.00039)
Supplement: Supplementary file 1 [file Data_Sheet_1.pdf]

Supplementary Material

1 SELECTING THE BEST CLASSIFIER

In the main manuscript support vector machine (SVM) was used as classifier for comparison of textural or pointillist feature spaces. Several additional types of classifier were also tested including decision tree, logistic regression and K-nearest neighbours (KNN) Bishop (2006). Classification performance for each of these classifiers coupled with textural and pointillist feature spaces were computed for synthetic data with all PSF sizes and for real data images. Tables S1, S2, S3, S4 and S5 provide the performances of the classification between (C_1,C_2) based on density differences respectively for auto correlation, GLCM, LBP, distance distribution and Ripley's $\hat{K}-$function feature spaces. Also, Table S6 shows the classification performances between two classes of real cells (healthy and unhealthy shown in Figure 2A of the main manuscript) using the four classifiers based on the same textural or pointillist feature spaces. On synthetic data no significant systematic gap of performances were found between the four tested classifiers when screened on a large range of PSF sizes. On real data the classifier based on a support vector machine (SVM) with a linear kernel provided the highest classification performances. This is why it was selected for the comparison of the feature spaces in the main manuscript.

Table S1. Classification accuracy (in percent %) for the discrimination between cells (C_1,C_2) populations based on density differences depending on the image resolution (PSF size, ratio k). Auto-correlation textural feature space coupled with four classifiers. The best classifier for each ratio k value of Eq. (1) is mentioned in bold.

.

**Classifiers**	**Classification performance (%) function of Ratio k**										
	0.1	*0.2*	*0.3*	*0.4*	*0.5*	*0.7*	*0.9*	*1.3*	*1.5*	*1.8*	*2*
Decision tree	93.8	91.2	93	91	91.2	89.8	87.8	87	86	88.4	89.6
Logistic regression	**94.4**	92.6	**93.8**	92.2	**94**	**92.4**	**90.4**	89.2	89.6	**89.2**	90
SVM	**94.4**	**93**	93.2	**92.6**	92.8	91.2	88.8	**89.8**	**90.4**	88.4	**90.2**
KNN	94	91	92.2	91.6	91.8	91.4	89.4	87.8	87.4	88	89.4

Table S2. Same as Table S1 but with GLCM textural feature space.

**Classifiers**	**Classification performance (%) function of Ratio k**										
	0.1	*0.2*	*0.3*	*0.4*	*0.5*	*0.7*	*0.9*	*1.3*	*1.5*	*1.8*	*2*
Decision tree	67.2	73	82	85.8	83.8	86.6	92	89.8	89.8	**91.8**	**94.6**
Logistic regression	**72.4**	**84.6**	87.4	**88.2**	**88.6**	**89.2**	**92.8**	**92.2**	89.8	91.4	94
SVM	72.2	**84.6**	**87.8**	88	**88.6**	88.4	92.2	91.6	90.2	91	94.2
KNN	68.6	81.8	85.8	86.4	85.6	87.2	90	89.6	**91.4**	89.8	93.2

2 OPTIMIZATION FOR GLCM AND LBP TEXTURAL METHODS

Two main parameters have to be tuned for best classification performance in both GLCM (Haralick et al. (1973)) and LBP (Ojala et al. (2002)) textural methods. These parameters are the size of the neighborhood for GLCM and the size of window in which images are divided during computation of LBP features. For optimization, classification performances based on GLCM and LBP textural methods were computed

Table S3. Same as Table S1 but with LBP textural feature space.

**Classifiers**	**Classification performance (%) function of Ratio k**										
	0.1	*0.2*	*0.3*	*0.4*	*0.5*	*0.7*	*0.9*	*1.3*	*1.5*	*1.8*	*2*
Decision tree	76.4	72	60.6	63.2	77.8	89.6	**92.4**	89.2	91.2	90.4	93
Logistic regression	**78.6**	78.4	63.4	**67.8**	**81.2**	91.6	**92.4**	**91.8**	**92.8**	**92.2**	93.4
SVM	78.4	**79**	**64.2**	**67.8**	81	**91.8**	**92.4**	91.2	92.4	**92.2**	**93.8**
KNN	77.2	77.8	56.4	62.4	79.2	90.4	90.4	88.4	90.8	91.2	92

Table S4. Same as Table S1 but with distance distribution feature space feature space.

**Classifiers**	**Classification performance (%) function of Ratio k**										
	0.1	*0.2*	*0.3*	*0.4*	*0.5*	*0.7*	*0.9*	*1.3*	*1.5*	*1.8*	*2*
Decision tree	67	58	**61.4**	54.6	65.2	57	62.6	81.6	84.6	90.6	85
Logistic regression	69.6	62.2	57	**59.8**	**77.2**	**66**	**65.6**	**87.2**	**84.8**	**93.4**	**91.2**
SVM	**70.2**	62.6	57.2	59	76.6	64.8	62.8	86.2	**84.8**	92.2	89.8
KNN	63.4	**63.6**	55.8	56.2	71.2	58	57.8	83.2	82.2	89.6	88.8

Table S5. Same as Table S1 but with Ripley's-$\hat{K}$ function feature space.

**Classifiers**	**Classification performance (%) function of Ratio k**										
	0.1	*0.2*	*0.3*	*0.4*	*0.5*	*0.7*	*0.9*	*1.3*	*1.5*	*1.8*	*2*
Decision tree	56.4	52.4	55.4	49.2	49	48.4	56.4	64.6	**67.8**	68.2	64.4
Logistic regression	**59.2**	53.8	56.4	48.2	46.4	**50.2**	55.2	**68**	65.4	**71.8**	65
SVM	59	53.2	**56.6**	**50.6**	48.6	48	**59.4**	67.2	65	**71.8**	**65.6**
KNN	54.6	**55.2**	50	47.8	**53.6**	49.6	55.8	64.4	65.2	67	62

Table S6. Classification results (in percent %) between real healthy and unhealthy cell populations. Four classifiers are used to study textural and pointillist feature spaces. The best classifier for each feature space is mentioned in bold.

**Feature spaces**	**Classifiers**			
	Decision tree	*Logistic regression*	*SVM*	*KNN*
Auto correlation	94.4	94.6	**94.7**	**94.7**
GLCM	62.7	68.7	**70**	67.3
LBP	**65.8**	64	64	62
Distances distribution	71.8	74.4	78.8	**79.8**
Ripley's $\hat{K}$-function	67.6	67.7	**69**	66.6

for the 11 σ_{psf} also used in Table S1 to S5 ranging from sub-resolved to super-resolved. A linear SVM classifier was used as classifier and feature spaces of LBP and GLCM were reduced to 5 using principal component analysis. Performances obtained for all PSF sizes were averaged. The size of the neighborhood and the size of window that gave the best classification results was found to be optimal at 72×72 (see figure S1).

3 SIMULATION BASED ON FLUORESCENT MARKERS DENSITY DIFFERENCES

In addition to the simulation presented in the main manuscript, we generated another two populations of cells mimicking healthy (C_1) and pathological (C_2) with a difference of marker density and without taking into account the diffraction effect of the microscope. The coordinates (x, y) of each fluorescent marker were

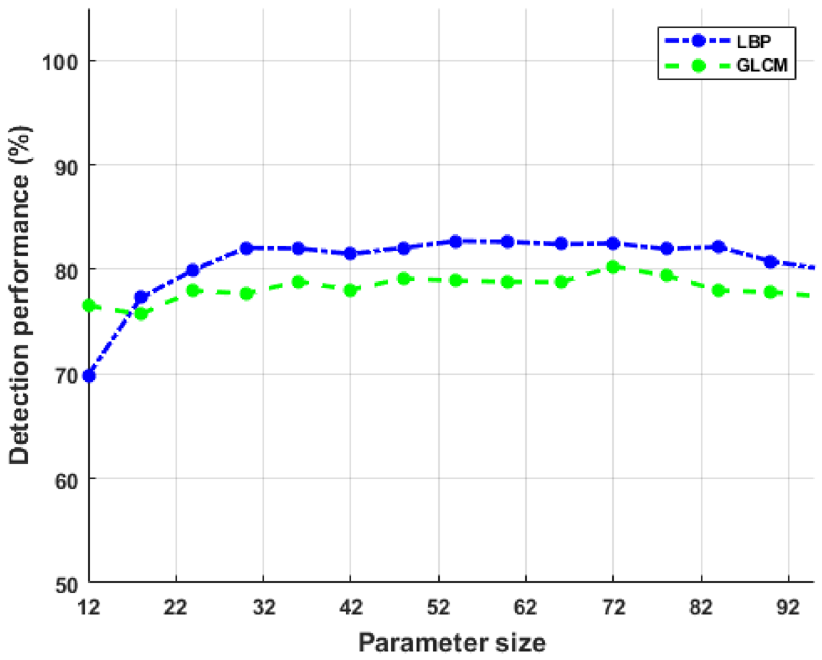

Figure S1. Classification performances averaged for all σ_{psf} as a function of the size of neighborhood and the size of window for GLCM and LBP respectively.

picked randomly according to independent and identically distributed Gaussian distributions on horizontal and vertical dimensions of an image of $M \times N$ pixels respectively, where $x \in \{1, 2, 3, ..., M = 256\}$ and $y \in \{1, 2, 3, ..., N = 256\}$. The two classes of cells where generated with for healthy cells (C_1) : $N_x(126, 100)$, $N_y(126, 100)$, a total amount of markers of 3000, and a resulting density of markers of 0.3. For the pathological cells (C_2) we have $N_x(126, 100)$, $N_y(126, 100)$, a total number of markers of 3100 and a resulting density of markers of 0.31. The difference of marker density between classes is of 0.01. We used here this difference between (C_1) and (C_2) to compare the pointillist and textural approaches.
After, sub and super resolved microscopy images are obtained by convolving the generated fluorescent dots with 11 various σ_{psf} sizes the same in the main manuscript.
Textural and pointillist feature spaces are studied and the performance of detecting the difference based on density is shown in figure S3. Globally, textural performances shows the same behavior as found in the case studied in the main manuscript. For pointillist approach, since the only difference between (C_1) and $C_2)$ is in the density, as expected no difference in the Ripley's $\hat{K}-$function are found as shown in Figure S2. However, when convolution and the UNLOCK detection are added some discriminant effect can occur between (C_1) and (C_2). Moreover, performances in distance distribution case is strongly affected by UNLOC efficiency that depend on the σ_{psf} size.

REFERENCES

Bishop, C. M. (2006). *Pattern recognition and machine learning* (springer)

Haralick, R. M., Shanmugam, K., et al. (1973). Textural features for image classification. *IEEE Transactions on Systems, Man, and Cybernetics* 3, 610 – 621

Ojala, T., Pietikäinen, M., and Mäenpää, T. (2002). Multiresolution gray-scale and rotation invariant texture classification with local binary patterns. *IEEE Transactions on Pattern Analysis & Machine*

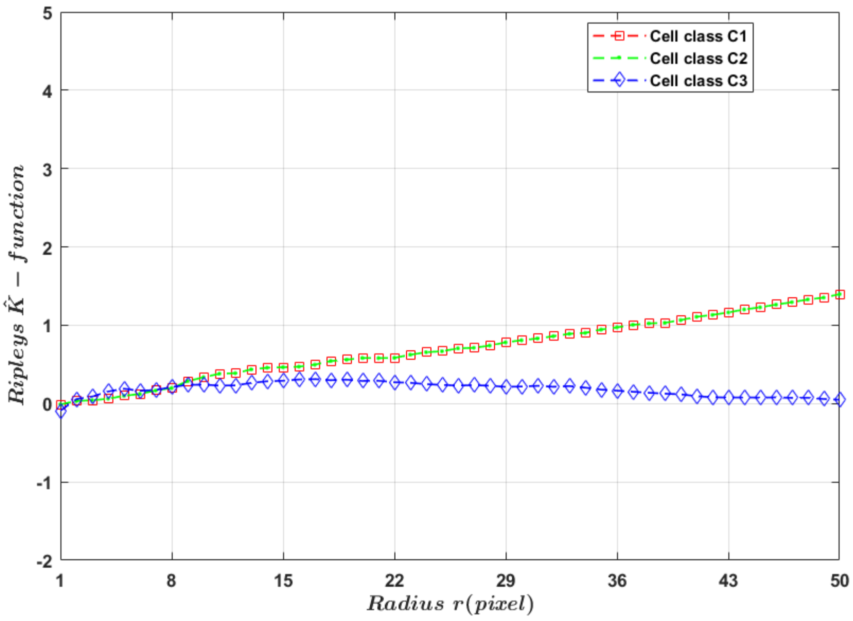

Figure S2. $\hat{K}(r,n)$ curve for raw markers (i.e. non convolved with a PSF) distribution according to radius r.

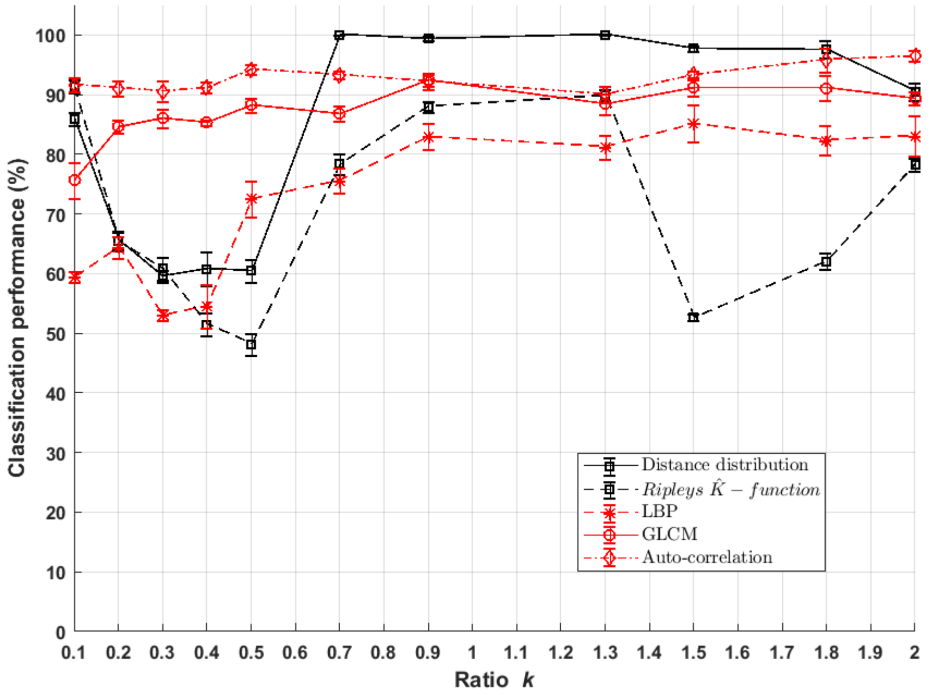

Figure S3. Performance of binary classification for marker density difference between cell classes C_1 and C_2 as a function of parameter k of Eq. (1) for both textural and pointillist approaches. The higher k the smaller the PSF size. Standard deviation of performances are computed using 10-folds cross-validation method. In red, methods following the textural approach, and, in black, methods following a pointillist approach.

Intelligence 24, 971 – 987
